# Hydrocarbon degradation potential and plant growth-promoting activity of culturable endophytic bacteria of *Lotus corniculatus* and *Oenothera biennis* from a long-term polluted site

**DOI:** 10.1007/s11356-017-9496-1

**Published:** 2017-07-06

**Authors:** Małgorzata Pawlik, Barbara Cania, Sofie Thijs, Jaco Vangronsveld, Zofia Piotrowska-Seget

**Affiliations:** 1 Department of Microbiology, University of Silesia, Katowice, Poland; 2 Research Unit Environmental Genomics, Helmholtz Zentrum München, Munich, Germany; 3 Environmental Biology, Centre for Environmental Sciences, Hasselt University, Hasselt, Belgium

**Keywords:** Plant-bacteria interactions, Endophytic bacteria, Petroleum hydrocarbons, Plant growth-promoting mechanisms, *Lotus corniculatus* L., *Oenothera biennis* L.

## Abstract

Many endophytic bacteria exert beneficial effects on their host, but still little is known about the bacteria associated with plants growing in areas heavily polluted by hydrocarbons. The aim of the study was characterization of culturable hydrocarbon-degrading endophytic bacteria associated with *Lotus corniculatus* L. and *Oenothera biennis* L. collected in long-term petroleum hydrocarbon-polluted site using culture-dependent and molecular approaches. A total of 26 hydrocarbon-degrading endophytes from these plants were isolated. Phylogenetic analyses classified the isolates into the phyla Proteobacteria and Actinobacteria. The majority of strains belonged to the genera *Rhizobium*, *Pseudomonas*, *Stenotrophomonas*, and *Rhodococcus*. More than 90% of the isolates could grow on medium with diesel oil, approximately 20% could use *n*-hexadecane as a sole carbon and energy source. PCR analysis revealed that 40% of the isolates possessed the *P450* gene encoding for cytochrome P450-type alkane hydroxylase (CYP153). In in vitro tests, all endophytic strains demonstrated a wide range of plant growth-promoting traits such as production of indole-3-acetic acid, hydrogen cyanide, siderophores, and phosphate solubilization. More than 40% of the bacteria carried the gene encoding for the 1-aminocyclopropane-1-carboxylic acid deaminase (*acdS*). Our study shows that the diversity of endophytic bacterial communities in tested plants was different. The results revealed also that the investigated plants were colonized by endophytic bacteria possessing plant growth-promoting features and a clear potential to degrade hydrocarbons. The properties of isolated endophytes indicate that they have the high potential to improve phytoremediation of petroleum hydrocarbon-polluted soils.

## Introduction

Over the last decades, plant-endophytic bacteria associations have received considerable attention. These associations play essential roles in beneficial interactions among plants and bacteria, and contribute to the ecological balance between them (Hardoim et al. 2008; Khan et al. 2013; Santoyo et al. 2016). Endophytes reside in plant tissues without any noticeable harmful effects on their host, and numerous studies have illustrated their positive effects on plant growth and development (Gaiero et al. 2013; Khan et al. 2013; Weyens et al. 2009d). As a consequence of their innate plant growth-promoting capabilities, they can contribute to a better adaptation of their host to biotic and abiotic stresses; facilitate the availability of nitrogen, phosphorus, and iron; produce phytohormones; and protect the plant against pathogens (Abbamondi et al. 2016; Compant et al. 2005; Glick et al. 2007; Naveed et al. 2014; Santoyo et al. 2016; Qin et al. 2015). Moreover, endophytes increase the tolerance of plants to contaminants by their degradation and/or detoxification, and reducing plant stress by production of 1-aminocyclopropane-1-carboxylic acid (ACC) deaminase (Barac et al. 2004; Gaiero et al. 2013; Huang et al. 2005; Ryan et al. 2008). Therefore, endophytic bacteria were claimed to be of significant importance in effective phytoremediation programs (Barac et al. 2009; Weyens et al. 2009a, b, c, d).

Phytoremediation is a green technology that utilizes plants and their associated microorganisms to clean up polluted soils and (ground)waters, and is considered a promising economical feasible strategy for the removal of organic contaminants from soils (Vangronsveld et al. 2009; Weyens et al. 2009c). Non-regulated disposal of oil sludge, oil extraction, refining, and leakage during storage and transport led to significant soil contamination and accumulation of aliphatic and aromatic hydrocarbons in the environment. Because many petroleum hydrocarbons can exert harmful effects on living organisms, removal of these organic pollutants has been intensively investigated (Huang et al. 2005; Khan et al. 2013; Wang et al. 2008). Recent evidences suggest that a key element for successful phytoremediation is the use of plants that tolerate high levels of contaminants, in combination with beneficial plant-associated endophytic bacteria capable of degrading pollutants (Germaine et al. 2009; Yousaf et al. 2010a, b; Yousaf et al. 2011). Furthermore, it has been shown that plants growing in hydrocarbon-contaminated soil can selectively enhance the prevalence of favorable endophytes containing genes encoding for enzymes responsible for hydrocarbon degradation (Oliveira et al. 2014; Siciliano et al. 2001). Also, other authors demonstrated that the presence of hydrocarbon pollution has led to increased numbers of endophytes with hydrocarbon degradation capabilities (Barac et al. 2009; Oliveira et al. 2014; Taghavi et al. 2009). It appears that exploring the diversity of the plant endophytic community is important for the selection of useful strains for phytoremediation of soils polluted with organics, improving plant adaptation and growth. Due to the broad range of symbiotic interactions between the plant host and its endophytes, their potential has great implications for biotechnological applications during phytoremediation (Khan et al. 2013; Li et al. 2012; Pilon-Smits 2005). Plant species capable of growing in soils heavily polluted with petroleum hydrocarbons make them a useful tool for phytoremediation purposes. Species used in this study are *Lotus corniculatus* (bird’s-foot trefoil) and *Oenothera biennis* (common evening-primrose), growing abundantly in a long-term crude oil polluted area in Silesia (Poland). Their high tolerance to hydrocarbon contamination may indicate that the tissues of these plants are colonized by hydrocarbon-degrading endophytes that will show more promise for detailed studies and applications. In recent studies, *Lotus* spp. were shown to possess a great potential to adapt to a number of abiotic stresses, making them suitable candidates for phytoremediation of degraded environments (Escaray et al. 2012; Yousaf et al. 2010a, b). Previously, alkane-degrading endophytic bacteria were isolated from *L. corniculatus* growing under laboratory conditions (Oliveira et al. 2014; Yousaf et al. 2010a). However, these studies were lacking important information about the composition of endophytic bacterial communities of *L. corniculatus* spontaneously colonizing heavily polluted environments. Also, much remains to be explored about the abundance and diversity of the endophytic bacteria associated with wild growing *O. biennis*. To the best of our knowledge, this is the first report describing the petroleum hydrocarbon-degrading endophytic bacteria of *O. biennis.*

Therefore, the aim of the study was to assess the potential of endophytes isolated from *L. corniculatus* and *O. biennis* for petroleum hydrocarbon degradation. Moreover, the composition of endophytic communities isolated from both plants and plant growth promotion potential of endophytes were investigated.

## Material and methods

### Isolation of endophytic bacteria and genotypic characterization

*L. corniculatus* (bird’s-foot trefoil) and *O. biennis* (common evening-primrose) were collected from a long-term polluted site located in Czechowice-Dziedzice, Silesia, Southern Poland (49° 54′ 49.0″ N, 19° 01′ 01.9″ E). The total petroleum hydrocarbons (TPH) content of the soil was 7460.5 ± 272.9 mg kg^−1^ dry soil (PN-EN ISO 16703:2011). The detailed soil characteristic was published previously (Pacwa-Płociniczak et al. 2016). Plants were sampled with soil adhering to the roots to a depth of 0–20 cm around a waste lagoon with acidic, highly weathered petroleum sludge. The plants were collected with adjusted soil in five replicates and transferred to the laboratory. The plants were kept in room temperature, and soil moisture was adjusted to constant level. During 5 days, the endophytic bacteria were isolated according the procedure described in this paper.

The plants were divided into roots, shoots, and leaves. Roots were shaken with 2% Tween 80 (water solution) to remove the adhering soil, and then transferred to a solution for surface sterilization. The pieces of roots were submerged in 0.01% active sodium hypochlorite, 10% hydrogen peroxide, 70% ethanol for 2 min, and rinsed three times with sterile distilled water. The stems and leaves were surface sterilized using 10% hydrogen peroxide, and 70% ethanol for 2 min, and rinsed three times with sterile distilled water. The sterilization efficiency was confirmed by plating 100 μL of last washing water onto a TSA medium. Subsequently, the roots, stems, and leaves were macerated with 0.9% NaCl.

Two approaches were used to isolate hydrocarbon-degrading endophytic bacteria. In the first strategy, the macerate of plant was directly plated on solid M9 mineral medium with crude oil (1%) as the sole source of carbon and energy. As a second method, liquid enrichment cultures were used. The macerated plant tissue was added to 100 mL liquid M9 mineral medium containing crude oil at the concentration of 1%. The flasks were incubated with shaking (120 rpm) for 7 days at 28 °C. Then, the enrichment culture was spread on a solid M9 mineral medium with crude oil (1%). All plates were incubated for 7 days at 28 °C. Morphologically distinct bacterial colonies were selected, purified, and stored at −20 °C.

From each isolated endophytic strain, DNA was extracted using the DNeasy Blood and Tissue Kit (Qiagen, Venlo, Netherlands). The 27F (5′ AGAGTTTGATCCTGGCTCAG 3′) and 1392R (5′ ACGGGCGGTGTGTRC 3′) primers were used for the PCR amplification of the 16S rRNA gene. The PCR master mix consisted of: DNA template, 1× High Fidelity PCR buffer (Invitrogen, Carlsbad, CA, USA), 0.2 mM L^−1^ of dNTP, 2 mM L^−1^ MgCl_2_, 0.2 μM L^−1^ each of the forward and reverse primers, and 1 U of High Fidelity Platinum Taq DNA polymerase (Invitrogen, Carlsbad, CA, USA) per 50 μL. The PCR conditions were denaturation at 94 °C for 5 min, 30 cycles of 94 °C for 1 min, 54 °C for 45 s, and 72 °C for 1.5 min, followed by final extension of 10 min at 72 °C. The PCR products (20 μL) were digested for 2 h at 37 °C with 1 U of the four-base-specific restriction endonuclease HpyCH4 IV in 1× NEB buffer (New England Biolabs). Amplified ribosomal DNA restriction analysis (ARDRA) was performed using obtained digestion fragments. They were separated and visualized by electrophoresis in a 1.5% agarose gel with GelRed (VWR, Leuven, Belgium). The same ARDRA banding patterns were grouped and representative patterns were selected for sequencing. The obtained 16S ribosomal DNA (rDNA) sequences from Macrogen (Amsterdam, the Netherlands) were compared with nucleotide sequences in the National Center for Biotechnology Information (NCBI) database using the Basic Local Alignment Search Tool (BLAST). A phylogenetic tree was constructed with the neighbor-joining method by the software MEGA 6.0. Bootstrap analysis was performed based on 1000 replicates. The sequences of 16 selected isolates were submitted to the NCBI GenBank database under accession numbers from KU726257 to KU726272.

### Plant growth-promoting and other characteristics

The ability of isolates to solubilize inorganic phosphate was tested using Pikovskaya agar medium. After 7 days of incubation at 28 °C, the presence of clear halos around colonies indicated a positive reaction, and considered as resulting from the utilization of tricalcium phosphate (Pikovskaya 1948).

The indole-3-acetic acid (IAA) production was estimated by a colorimetric method using Salkowski’s reagent as described by Patten and Glick (2002). Concentration of IAA produced was estimated against standard curve of IAA in the range of 10–100 μg mL^−1^. Hydrogen cyanide (HCN) production was examined by adapting the method of Lorck (1948). Bacteria were cultured on nutrient broth (NB) supplemented with glycine at the concentration of 4.4 g L^−1^ at 28 °C for 4 days. Then bacteria were plated on the same solid medium and a Whatman filter paper soaked in solution of 2% Na_2_CO_3_ in 0.5% picric acid was placed on top of the medium. Plates were sealed with parafilm and incubated at 28 °C for 7 days. Production of HCN was indicated by color change of filter paper from yellow to orange-brown.

Siderophore release was evaluated on a Chrome Azurol S (CAS) agar medium as proposed by Schwyn and Neilands (1987). A positive reaction was considered when the color of CAS medium was changed from blue to yellow or orange.

The ACC deaminase gene *acdS* was PCR-amplified using primers F1936 (forward) 3′ GHGAMGACTGCAAYWSYGGC 5′, F1938 (reverse) 3′ ATCATVCCVTGCATBGAYTT 5′, F1939 (reverse) 3′ GARGCRTCGAYVCCRATCAC 5′ (Blaha et al. 2006). The PCR mixture and conditions were prepared as described before (Kukla et al. 2014; Pawlik and Piotrowska-Seget 2015).

To determine the cellulase production, the bacterial cultures were plated on medium with carboxymethyl cellulose as the sole carbon source. After 7 days of incubation at 28 °C, the Lugol’s iodine was spread on the plate surface and yellow zones formed around the colonies indicated positive results (Pointing 1999).

To assess the swarming motility of bacterial strains, a semi-solid 0.2% NB agar was used. A diffuse zone of growth around the inoculation site at the center of the plate was considered as a positive result. The halo zone of growth was observed macroscopically after 24–48 h of incubation at 28 °C.

In all plant growth-promoting (PGP) tests, *Enterobacter intermedius* MH8b and *Escherichia coli* DH5α were used as the positive and negative controls, respectively (Płociniczak et al. 2013).

The emulsification activity was determined using the emulsification index (E24). The isolated endophytic strains were cultured on LB broth, M9 mineral medium with crude oil (1%), or *n*-hexadecane (1%) at 120 rpm on a rotary shaker. After 7 days at 28 °C, the cultures were centrifuged (10,600×*g*, 5 min) and 2 mL of supernatant were vigorously vortexed with 3 mL of substrate (diesel oil, *n*-hexadecane, or *para*-xylene). After 24 h, the height of the emulsion was measured. The E24 was presented as a percentage of height of the emulsified layer (mm) divided by total height of the liquid column (mm). In all measurements, the obtained results were compared with a positive control. This positive control was prepared by vortexing 2 mL of 5% solution of SDS (sodium dodecyl sulfate) and 3 mL of substrate (diesel oil, *n*-hexadecane, or *para*-xylene).

### Potential degradation of hydrocarbons and hydrocarbon-degradation genes

The ability to use petroleum hydrocarbons by isolated bacteria as a sole carbon and energy source was checked on a 40-mL liquid M9 mineral medium containing 1% of crude oil, diesel oil, *n*-hexadecane, or *para*-xylene. No other carbon source was added to the medium, and each treatment was performed with three replicates. The strains were incubated at 28 °C for 7 days, and their growth was compared with overall growth on NB. The growth kinetic in liquid media was performed to evaluate the growth of the strains with hydrocarbons as a sole carbon source. Results were considered positive when the optical densities of M9 and NB were similar.

The isolates were analyzed for the presence of specific genes encoding enzymes involved in hydrocarbon degradation, e.g., *alkB* (alkane monooxygenase), *alkH* (alkane hydroxylase), *P450* (cytochrome P450-type alkane hydroxylase, CYP153), *C23O* (catechol-2,3-dioxygenase), and *pah* (alpha subunit of the PAH-ring hydroxylating dioxygenases). DNA extraction and PCR conditions were described previously (Kukla et al. 2014).

## Results

### Isolation of endophytic bacteria and genotypic characterization

Collected plants were growing in soil containing high levels of petroleum hydrocarbons (7460.5 ± 272.9 mg kg^−1^ dry soil). In order to obtain hydrocarbon-degrading endophytic bacteria, only endophytes able to grow on M9 medium with 1% crude oil were isolated. Based on morphological characteristics 26 different endophytic strains were selected from roots, stems, and leaves of *L. corniculatus* and *O. biennis.*

**Fig. 1:**
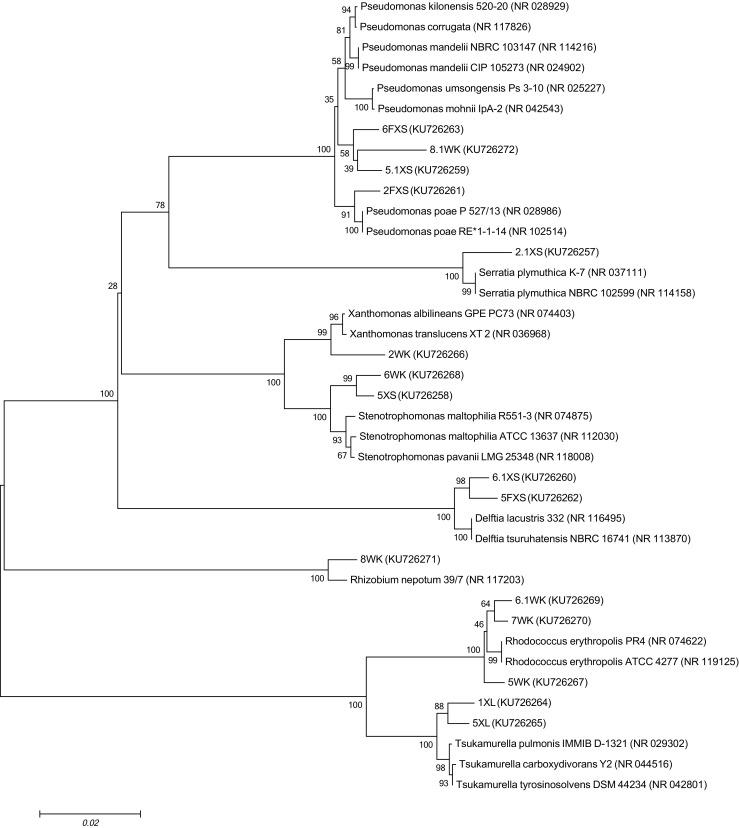
Neighbor-joining phylogenetic tree of partial 16S rDNA sequences of endophytic bacteria isolated from *L. corniculatus* and *O. biennis*. GenBank accession numbers of the strains are shown in *parentheses*. The *bar* represents 0.02 substitutions per site; bootstrap values (*n* = 1000) are displayed. The tree was generated using the MEGA 6.0 software

The isolates were identified based on sequencing of their 16S rDNA genes. To compare the genetic diversity, strains were grouped according to their ARDRA profiles (Table 1). Therefore, 16 strains presenting different ARDRA patterns were sequenced and identified by BLAST (similarities of ≥98%), and a phylogenetic tree was constructed (Fig.1).

**Table 1:**
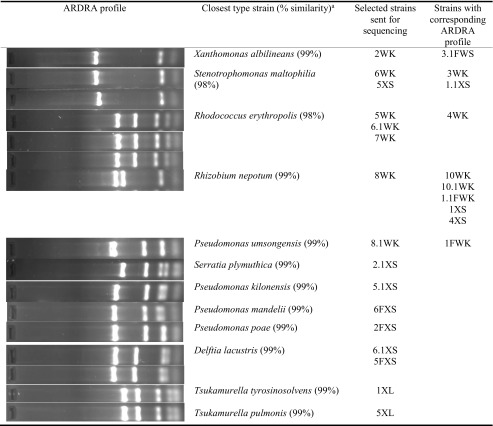
Based on ARDRA profiles, 16 bacteria represented different clusters of patterns were selected and identified based on 16S rDNA gene sequencing

^a^Identification based on 16S rRNA gene sequencing

**Fig. 2:**
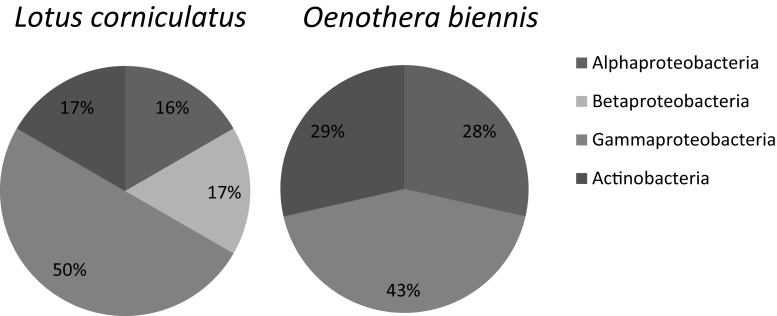
The graphs showing the classification of *L. corniculatus* and *O. biennis* endophytic bacteria to the Alpha-, Beta-, Gammaproteobacteria, or Actinobacteria. The percentages indicate the relative abundance of isolates that were present in the tested plants

Alphaproteobacteria, Gammaproteobacteria, and Actinobacteria were observed in both plant species. However, Betaproteobacteria were found only in *L. corniculatus* and were represented by *Delftia* sp. Gammaproteobacteria comprised the majority of the isolated strains and were the predominant group in both investigated plant species (Fig. 2). Bacterial isolates belonging to the genera *Rhizobium* and *Rhodococcus* were the most abundant in tissues of *O. biennis*. *Pseudomonas* sp. was the most often isolated from *L. corniculatus.* Bacteria from the genera *Delftia*, *Serratia*, and *Tsukamurella* were only found in *L. corniculatus*. Strains belonging to the genera *Rhodococcus* and *Xanthomonas* were the most characteristic endophytic strains for *O. biennis*.

### Potential degradation of hydrocarbons and hydrocarbon-degradation genes

The isolated endophytic strains were investigated for their hydrocarbon degradation potential. Their ability to grow on media containing diesel oil, *n*-hexadecane, and *para*-xylene as sole carbon sources was evaluated. More than 90% of all isolates were able to grow on medium with diesel oil. The potential to use *n*-hexadecane as a source of energy and carbon was observed in 16.67 and 28.57% of the *L. corniculatus* and *O. biennis* associated strains, respectively. All *n*-hexadecane-degrading bacteria belonged to the genera *Rhodococcus* and *Tsukamurella*. None of tested strains could make use of *para*-xylene as a sole carbon source.

The isolated endophytic bacteria were also screened for the presence of hydrocarbon degradation genes. Only 5 out of the 26 tested strains, namely *Pseudomonas mandelii* 6FXS, and *Rhodococcus* sp. (4WK, 5WK, 6.1WK, 7WK) presented a positive PCR result with *alkB* primers. The appearance of a positive PCR result for the *alkH* gene was observed for six strains belonging to the genera *Tsukamurella* and *Rhodococcus*. The gene *P450* responsible for the production of cytochrome P450-type alkane hydroxylase (CYP153) was detected in 41.67 and 42.86% *L. corniculatus* and *O. biennis* associated strains, respectively. Further, none of tested strains possessed genes encoding for catechol-2,3-dioxygenase (*C23O*), and the alpha subunit of the PAH-ring hydroxylating dioxygenases (*pah*).

### Plant growth-promoting and other characteristics

All isolated endophytic strains were screened for their plant growth-promoting traits. The obtained results are summarized in Table 2.

**Table 2:** Identification and characterization of endophytic bacteria from *L. corniculatus* and *O. biennis*

			16S rRNA identity	PGP mechanisms				Other abilities		Genes^f^				Degradation potential		
Plant	Tissue	Strain	Closest type strain	PS^a^	IAA^b^	HCN^c^	SID^d^	CMC^e^	Motility	ACCD (*acdS*)	*alk B*	*alk H*	*P450*	Crude oil	Diesel oil	*n*-hexadecane
*Lotus corniculatus*	Stem	1XS	*Rhizobium* sp.	1.0 ± 0.0	35.9 ± 3.7	−	−	−	+	−	−	−	−	+	+	−
		1.1XS	*Stenotrophomonas* sp*.*	−	24.3 ± 0.9	+++	ng	−	+	−	−	−	−	+	−	−
		2.1XS	*Serratia plymuthica*	1.8 ± 0.5	5.8 ± 0.1	++	+	6.3 ± 1.2	+	−	−	−	−	+	+	−
		4XS	*Rhizobium* sp.	−	18.8 ± 0.6	++	−	−	+	+	−	−	−	+	+	−
		5XS	*Stenotrophomonas maltophilia*	−	22.1 ± 1.5	+++	ng	−	+	+	−	−	+	+	+	−
		5.1XS	*Pseudomonas kilonensis*	7.3 ± 0.6	12.0 ± 0.3	+	−	4.7 ± 0.6	+	+	−	−	+	+	+	−
		6.1XS	*Delftia lacustris*	−	59.9 ± 4.0	−	+	−	+	−	−	−	+	+	+	−
		2FXS	*Pseudomonas poae*	10.3 ± 1.5	7.9 ± 0.3	+++	+	−	+	−	−	−	−	+	+	−
		5FXS	*Delftia lacustris*	−	62.6 ± 5.2	+	−	−	+	−	−	−	−	+	+	−
		6FXS	*Pseudomonas mandelii*	2.3 ± 0.6	16.5 ± 0.8	++	+	7.7 ± 1.5	+	−	+	−	−	+	+	−
	Leaf	1XL	*Tsukamurella tyrosinosolvens*	5.3 ± 0.6	2.0 ± 0.3	+	+	15.3 ± 2.1	−	+	−	+	+	+	+	+
		5XL	*Tsukamurella pulmonis*	5.67 ± 0.6	1.9 ± 0.2	++	+	14.7 ± 0.6	−	+	−	+	+	+	+	+
	Total (%)			58.33	100	83.33	50	41.67	83.33	41.67	8.33	16.67	41.67	100	91.67	16.67
*Oenothera biennis*	Root	2WK	*Xanthomonas albilineans*	−	17.2 ± 1.1	−	−	−	+	−	−	−	−	+	+	−
		3WK	*Stenotrophomonas* sp.	−	23.4 ± 2.0	+++	ng	−	+	+	−	−	−	+	+	−
		4WK	*Rhodococcus* sp.	−	1.8 ± 0.1	+	ng	2.3 ± 0.6	−	−	+	+	+	+	+	+
		5WK	*Rhodococcus erythropolis*	−	1.4 ± 0.1	−	ng	2.0 ± 0.0	−	−	+	+	+	+	+	+
		6WK	*Stenotrophomonas maltophilia*	−	18.6 ± 2.8	+++	ng	−	+	−	−	−	−	+	+	−
		6.1WK	*Rhodococcus erythropolis*	−	2.0 ± 0.2	+++	ng	2.7 ± 0.6	−	−	+	+	+	+	+	+
		7WK	*Rhodococcus erythropolis*	−	1.3 ± 0.0	+++	ng	1.7 ± 0.6	−	−	+	+	+	+	+	+
		8WK	*Rhizobium nepotum*	3.0 ± 0.0	15.3 ± 1.8	−	−	1.7 ± 0.6	+	+	−	−	−	+	−	−
		8.1WK	*Pseudomonas umsongensis*	4.0 ± 0.6	7.9 ± 1.2	+++	+	−	+	−	−	−	+	+	+	−
		10WK	*Rhizobium* sp.	−	27.5 ± 1.0	−	−	1.7 ± 0.6	+	+	−	−	−	+	+	−
		10.1WK	*Rhizobium* sp.	−	29.6 ± 1.0	++	−	2.0 ± 0.0	+	+	−	−	−	+	+	−
		1FWK	*Pseudomonas* sp.	3.0 ± 0.0	7.5 ± 0.2	−	+	2.0 ± 0.0	+	+	−	−	+	+	+	−
		1.1FWK	*Rhizobium* sp.	1.3 ± 0.6	22.5 ± 1.5	+++	−	3.0 ± 1.0	+	+	−	−	−	+	+	−
	Stem	3.1FWS	*Xanthomonas* sp.	−	16.3 ± 1.0	+++	−	−	+	−	−	−	−	+	+	−
	Total (%)			28.57	100	64.29	14.29	64.29	71.43	42.86	28.57	28.57	42.86	100	92.86	28.57

Determination of their potential plant growth - promoting traits, their production of cellulase, motility, degradation of hydrocarbons, and occurrence of genes encoding for enzymes involved in hydrocarbons degradation. ± standard deviation of three replicates

^a^Inorganic calcium phosphate solubilization, the halo diameter [mm]

^b^Indole-3-acetic acid production [μg mL^−1^ of IAA]

^c^Hydrogen cyanide production, − absent; + low efficiency; ++ medium efficiency; +++ high efficiency

^d^Siderophore production, − absent; + present; no growth (ng)

^e^Cellulase production, the halo diameter [mm]

^f^Detection of gene encoding for *acdS* enzyme ACCD (1-aminocyclopropane-1-carboxylate deaminase), *alkB* alkane monooxygenase, *alkH* alkane hydroxylase, *P450* cytochrome P450-type alkane hydroxylase, and CYP153

The test for tricalcium phosphate solubilization revealed that 58.33% of the *L. corniculatus* isolates could produce a halo zone on Pikovskaya medium. One strain, *Pseudomonas* sp. 2FXS, even produced a clear halo with more than 10 mm diameter. 28.57% of the endophytic strains from *O. biennis* showed able to solubilize phosphate in vitro but with low efficiency (1.3 ± 0.6 up to 3.0 ± 0.0 mm of clear zone).

All tested strains produced IAA in the presence of tryptophan. The IAA production ranged from 1.9 ± 0.2 up to 62.6 ± 5.2 μg mL^−1^ of IAA for isolates of *L. corniculatus* and from 1.3 ± 0.0 up to 29.6 ± 1.0 μg mL^−1^ of IAA for strains originating from *O. biennis*. The highest productions were found for three strains isolated from the stem of *L. corniculatus*: *Delftia lacustris* 5FXS, *Delftia lacustris* 6.1XS, and *Rhizobium* sp. 1XS, which produced 62.6 ± 5.2, 59.9 ± 4.0, and 35.9 ± 3.7 μg mL^−1^ of IAA, respectively. In comparison, strains *Rhodococcus erythropolis* 7WK, *Rhodococcus erythropolis* 5WK, and *Rhodococcus* sp. 4WK isolated from roots of *O. biennis* showed lower IAA production potentials of 1.3 ± 0.0, 1.4 ± 0.1, and 1.8 ± 0.1 μg mL^−1^ IAA, respectively.

The ability of the endophytic bacteria to control pathogens by producing HCN was tested. As shown in Table 2, respectively, 83.33 and 64.29% of the endophytes isolated from *L. corniculatus* and *O. biennis* could synthesize HCN.

Siderophore production was observed in higher numbers of *L. corniculatus*-associated endophytic strains (50%) (Table 2). Among the isolates from *O. biennis*, only for 14.29% a positive result was observed. Several strains could not grow on the CAS medium.

The ACC deaminase production potential of the strains was evaluated based on the PCR amplification of the *acdS* gene. A positive PCR result was obtained for, respectively, 41.67 and 42.86% of the strains isolated from *L. corniculatus* and *O. biennis* (Table 2).

The potential plant colonization abilities of the endophytic strains were evaluated based on their cellulase activity and motility. The percentages of cellulase producers among *L. corniculatus* and *O. biennis* isolates were 47.67 and 64.29%, respectively (Table 2). In this test, endophytes of *L. corniculatus* produced larger halo zones than those of *O. biennis* and were in the range of 4.7 ± 0.6 up to 15.3 ± 2.1 mm. With regard to their plant colonization capacities, all isolates were also screened using a swarming motility assay. 83.33% of the *L. corniculatus* associated strains and 71.43% of the strains isolated from *O. biennis* showed positive for motility.

Due to the hydrophobic nature of petroleum hydrocarbons, the endophytic strains were tested for their emulsification capacity which could be connected with biosurfactant production. Only six isolates (*Serratia plymuthica* 2.1XS, *Delftia lacustris* 6.1XS, *Rhodococcus* sp. 4WK, *Rhodococcus erythropolis* 6.1WK, *Rhizobium nepotum* 8WK, and *Pseudomonas umsongensis* 8.1WK) showed emulsification abilities (Table 3). *Rhodococcus* sp. 4WK demonstrated the highest emulsification capability against diesel oil (E24 = 52.78%), *n*-hexadecane (E24 = 52.78%), and *para*-xylene (E24 = 58.33%). The supernatant obtained after growing strain *R. erythropolis* 6.1WK on M9 medium with *n*-hexadecane, showed emulsification activities against diesel oil (E24 = 50%) and *para*-xylene (E24 = 50%), but not for *n*-hexadecane. In the case of other strains, emulsification capabilities were observed after their growth on the M9 medium with crude oil. These strains exhibited emulsification activity only against *para*-xylene (Table 3).

**Table 3:** Emulsification activities of endophytic bacteria

Strain	M9 with crude oil			M9 with *n*-hexadecane		
	Emulsification index (%)					
	D	H	X	D	H	X
*Serratia plymuthica* 2.1XS	–	–	41.67 ± 0.84	–	–	–
*Delftia lacustris* 6.1XS	–	–	41.67 ± 0.42	–	–	–
*Rhodococcus* sp*.* 4WK	52.78 ± 0.46	52.78 ± 1.41	58.33 ± 0.59	47.22 ± 0.39	47.22 ± 0.37	52.78 ± 0.92
*Rhodococcus erythropolis* 6.1WK	–	–	–	50 ± 0.53	–	50 ± 0.45
*Rhizobium nepotum* 8WK	–	–	47.22 ± 0.53	–	–	–
*Pseudomonas umsongensis* 8.1WK	–	–	47.22 ± 0.34	–	–	–

Strains were cultured in M9 mineral medium with one carbon source (crude oil or *n*-hexadecane). The values of emulsification index were determined for different substrates diesel oil, *n*-hexadecane, and *para*-xylene. Given are means ± standard deviation of three replicates

*D* diesel oil, *H n*-hexadecane, *X p*-xylene

## Discussion

The study of endophytic bacteria associated with plants spontaneously colonizing long-term petroleum hydrocarbon-polluted soils is of high significance. It appears that bacteria associated with these plants exhibit several beneficial traits involved in increasing the availability of nutrients, degradation of pollutants, and production of active metabolites and phytohormones (Andreolli et al. 2013; Ho et al. 2012; Porteous-Moore et al. 2006; Sun et al. 2014a, 2014b).

Our investigation of the culturable hydrocarbon-degrading endophytic bacteria associated with *L. corniculatus* and *O. biennis* indicated that the tissues of these plant species were mainly inhabited by strains belonging to the Gammaproteobacteria with a high predominance of *Pseudomonas* sp*.* (Fig. 2). Similar results were reported for the culturable bacterial endophytes isolated from *Solidago canadensis* collected in an oil area that had been in operation since the mid nineteenth century (Lumactud et al. 2016). Representatives of the genus *Pseudomonas* are frequently reported plant endophytes and are known for their great abilities to promote plant growth, degrade xenobiotic compounds, induce resistance in plants, or enhance phytoremediation (Compant et al. 2005; Gómez-Lama et al. 2014; Khan et al. 2013; Preston 2004; Sessitsch et al. 2013; Sun et al. 2014a).

In general, the culturable endophytic bacterial communities of *L. corniculatus* and *O. biennis* growing in petroleum hydrocarbons polluted soil were characterized by a low diversity. The isolated strains belonged to two main phyla: Proteobacteria and Actinobacteria (Tables 1 and 2). The domination of Proteobacteria in the endophyte assemblages of *Alopecurus aequalis* and *Oxalis corniculata* growing in soil polluted with different levels of polycyclic aromatic hydrocarbons (PAH) was also reported by Peng et al. (2013). Moreover, these authors also found that with increasing levels of PAH, the numbers of endophytic bacteria decreased rapidly. Interestingly, at the same PAH pollution level, the dominant endophytic bacteria in *A. aequalis* and *O. corniculata* were different (Peng et el. 2013). In our study, we observed a similar trend since *Pseudomonas* sp. dominated inside *L. corniculatus* whereas *Rhizobium* sp. constituted the majority of the culturable endophytes of *O. biennis*. However, *Delftia* sp., *Serratia* sp., and *Tsukamurella* sp. were only detected inside tissues of *L. corniculatus* while *Rhodococcus* sp. and *Xanthomonas* sp. were isolated only from *O. biennis.* It is remarkable that specific bacterial taxa colonize only specific plant species even if they are grown at the same conditions (Lumactud et al. 2016; Phillips et al. 2008). Phillips et al. (2008) showed that *Medicago sativa*, *Lolium perenne*, *Elymus angustus*, *Pucinella nuttalliana*, and *Agropyron elongatum* growing in weathered hydrocarbon contaminated soil had distinct endophytic microbial populations with potential to degrade various hydrocarbons. Plant species specificity showed also bacterial endophytic communities from *Achillea millefolium*, *Solidago canadensis*, *Trifolium aureum*, and *Dactylis glomerata* collected in area contaminated by hydrocarbons (Lumactud et al. 2016). It indicates that despite of hydrocarbons, which are a strong selection pressure, plant species remain a driving factor to endophytic communities (Lumactud et al. 2016). Studies by Tardif et al. (2016) showed that apart of plants, the level of petroleum hydrocarbon contamination appeared to be an important factor influencing the number and diversity of endophytes. They reported that increasing concentrations of hydrocarbons caused shift in endophytes, favoring putative contaminants degraders and microorganisms with potential to promote plant growth and health. Another key factor that shapes the structure of rhizospheric and also endophytic communities is composition of root exudates (Oliveira et al. 2012; Wael et al. 2014). Different plant species secrete different root exudates which may promote the growth and metabolic properties of microorganisms which are desired by the plants (Andreolli et al. 2013). However, studies on the molecular determinants and interactions in endophytic colonization as well as genetic basis for these processes are still lacking.

Endophytic bacteria can improve the removal of xenobiotics by decreasing the amounts of pollutants in soils (Barac et al. 2004; Germaine et al. 2009). The results of our study have proven that many bacterial endophytes have the potential to degrade various organic contaminants. More than 90% of the isolates were able to grow on diesel oil. Long-term coevolution between plants and their endophytic bacteria leads to the development of bacteria containing genes encoding hydrolytic enzymes involved in the decomposition of various compounds of plant origin. Several authors characterized endophytic bacteria for their capacities to detoxify BTEX compounds (benzene, toluene, ethylbenzene, and xylene), hydrocarbons such as phenanthrene, fluorene, naphthalene, trinitrotoluene, and PAH (Andreolli et al. 2013; Oliveira et al. 2014; Phillips et al. 2008; Taghavi et al. 2009; Thijs et al. 2014; Yousaf et al. 2010a, 2010b). In this study, many endophytic bacteria exhibited the potential to degrade crude oil, diesel oil, and/or *n*-hexadecane. The occurrence of *Rhodococcus* sp. and *Pseudomonas* sp. was interesting due to their already often described abilities for plant growth promotion and hydrocarbon degradation (Kukla et al. 2014; Pawlik and Piotrowska-Seget 2015). These strains were repeatedly reported as organic pollutant degraders (Porteous-Moore et al. 2006; Zampolli et al. 2014). Our previous studies showed that the dominant cultivable endophytic bacteria inhabiting ryegrass and tall hawkweed growing in petroleum contaminated soil also belonged to the genus *Pseudomonas*. Members of *Rhodococcus* genus were also isolated although not in high abundance (Kukla et al. 2014; Pawlik and Piotrowska-Seget 2015).

Almost half of the isolated strains were equipped with the cytochrome P450 hydroxylase gene (CYP153) involved in degradation of short and medium chain-length *n*-alkanes and fatty acids (van Beilen et al. 2006). According to this result, endophytic bacteria may metabolize aliphatic hydrocarbons in plants. On the other hand, various bacteria with genes encoding alkane-degrading enzymes have been found in different environments originally not contaminated with hydrocarbons which suggest also other roles for *alkB* and/or *P450* (CYP153) genes. For example, plant envelopes consist of cuticular waxes composed of long-chain alkanes and by consequence, the ability to degrade alkanes is considered an important first step for endophytic bacteria to enter the plant (Nie et al. 2014; van Bogaert et al. 2011).

One of the limiting factors in bacterial degradation of petroleum hydrocarbons is their hydrophobicity (Kosaric 2001; Pacwa-Płociniczak et al. 2011). Some bacteria produce biosurfactants, increasing the bioavailability of hydrophobic molecules to microorganisms and their further biodegradation. In this study, biosurfactant producers belonged to the genera *Serratia*, *Delftia*, *Rhodococcus*, *Rhizobium*, and *Pseudomonas*. *Rhodococcus* sp. 4WK showed able to emulsify diesel oil, *n*-hexadecane, and *para*-xylene with high efficiency (Table 3). These emulsification capabilities suggest that these endophytic strains may contribute to the removal of petroleum hydrocarbons by increasing their accessibility. However, due to the different structures of biosurfactants, they may play diverse roles in nature. They can inhibit the growth of pathogen, support colonization of plant tissue, improve motility, and improve physical access of enzymes involved in cell wall degradation to the plant surface (Andersen et al. 2003; Raaijmakers et al. 2006; Velho et al. 2011). Hence, biosurfactant production may be another mechanism important in endophytic behavior.

The cellulase secretion and motility of endophytic bacteria may help in the process of plant colonization and spread of bacteria in the host plant. Verma et al. (2001) and Pereira et al. (2016) described the cell wall-degrading enzymes from endophytic bacteria, demonstrating their potential for plant tissues colonization. The ability of bacterial strains to colonize roots appears to be crucial for a close interaction between plants and bacteria especially in hazardous environments. Germaine et al. (2009) suggested that the low ability of *Pseudomonas putida* G7 to colonize roots might explain the weak phytoprotection effect of this strain in a naphthalene-polluted soil. On the other hand, the study of Andreolli et al. (2013) demonstrated that the PAH-degrading *Burkholderia fungorum* DBT1 might colonize roots that increased phytoremediation efficiency of PAH. In our study, cellulase production was detected in a high number of endophytic strains (41.67% *L. corniculatus*, 64.29% *O. biennis*), suggesting that this trait is closely related to endophytic behavior.

Bacterial strains with PGP capabilities have important functions for plants growing in stressful environments polluted with hazardous compounds. For their further use in phytoremediation studies, strain selection should be based on their potential to promote plant growth and hydrocarbon degradation.

The most important PGP mechanism of endophytic bacteria seems to be IAA production. All strains that we isolated were IAA producers (Table 2). Similarly, other authors observed that a vast majority of endophytic bacteria synthesized IAA (Etesami et al. 2014; Kukla et al. 2014; Pawlik and Piotrowska-Seget 2015; Pereira et al. 2016; Xu et al. 2016). The application of these strains could contribute to improve root proliferation and elongation that is crucial during phytoremediation as well as in growth and development of economical important crops such as potatoes, maize, and rice (Garbeva et al. 2001; Ikeda et al. 2010; Seghers et al. 2004). An increased formation of root hairs was observed in *Arabidopsis thaliana* seedlings after inoculation with all IAA producing endophytic strains while from the rhizospheric strains, only 50% induced a similar effect (Abbamondi et al. 2016). Furthermore, strains with the ability to produce IAA seem to better colonize plant roots than other strains. Etesami et al. (2014) reported a strong correlation between IAA production by bacteria and their plant colonization efficiency. IAA-producing endophytic strains colonized rice seedlings better in comparison with soil bacteria. However, both positive and negative effects of IAA on plant roots development and growth have been reported (Zúñiga et al. 2013). This is linked with the amounts of IAA produced by the bacteria. The high IAA producer strain *Pantoea* sp. FF34 induced less root biomass in comparison with strains produced markedly lower concentrations of IAA (Naveed et al. 2014).

Strains possessing ACC deaminase activity attract much attention as plant growth-promoting bacteria. This enzyme utilizes the precursor of ethylene that results in the decreasing of plants ethylene concentration and limiting its negative influence on plant growth (Glick et al. 2007; Hardoim et al. 2008). Etesami et al. (2014) observed a significant relationship among IAA and ACC deaminase production for endophytic bacteria which makes them suggesting that these PGP traits may be critical for endophytic and rhizospheric competence. Indeed, in the present study, almost half of isolates showed the potential to produce ACC deaminase as demonstrated by the positive PCR results (*acdS* gene). Plants growing in contaminated soils poor in nutrients need rhizospheric and endophytic bacteria that are able to produce phytohormones and improve the nutrient status of the host (Thijs et al. 2014). ACC deaminase-producing bacteria may reduce stress symptoms, stimulate the proliferation of roots, and promote the development of an extensive root system. These activities may increase contaminant uptake and their further biodegradation by plants or other bacteria (Arshad et al. 2007; Qin et al. 2015). For example, strain *Pantoea* sp. BTRH79 which possesses ACC deaminase activity improved plant fitness and growth in the presence of diesel contaminants (Arslan et al. 2014). *Burkholderia phytofirmans* PsJN with a deleted fragment of the *acdS* gene lost its ACC deaminase activity and ability to promote roots growth of canola (Sun et al. 2009).

## Conclusion

In order to exploit endophytic bacteria for improving phytoremediation efficiency, they should be well characterized. A better knowledge of these bacteria may allow utilizing them to increase remediation polluted soils. Plant growth-promoting and hydrocarbon-degrading endophytic bacterial strains were isolated from the plant species *L. corniculatus* and *O. biennis.* Our study indicated that all strains tested positive for at least one PGP trait and possessed genes encoding for the hydrocarbon degradation enzymes. Several strains were able to grow in the presence of crude oil, diesel oil, and to a lesser-extent *n*-hexadecane. In addition, our data enable to select endophytic bacterial strains which could enhance phytoremediation of hydrocarbon-polluted sites.
